# Embrace Open, Collaborative, Discovery-Based Exposomics

**DOI:** 10.1021/acs.est.6c02651

**Published:** 2026-06-26

**Authors:** Emma L. Schymanski, Foteini Raptopoulou, Vasiliki Gkanali, Roel Vermeulen

**Affiliations:** † Luxembourg Centre for Systems Biomedicine (LCSB), University of Luxembourg, 6 av du Swing, L-4367 Belvaux, Luxembourg; ‡ Julius Center for Health Sciences and Primary Care, University Medical Center Utrecht, Utrecht University, 3584 CS Utrecht, The Netherlands; § Institute of Risk Assessment Sciences, Utrecht University, 3584 CS Utrecht, The Netherlands

**Keywords:** transformation products, exposomics, chemical space

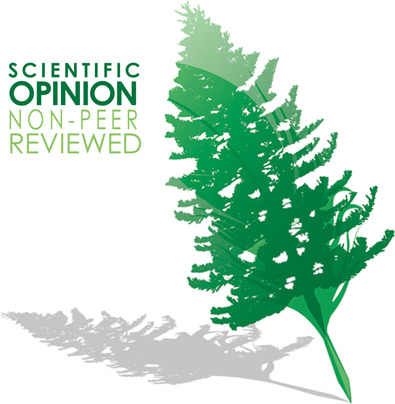

## Graphical abstract

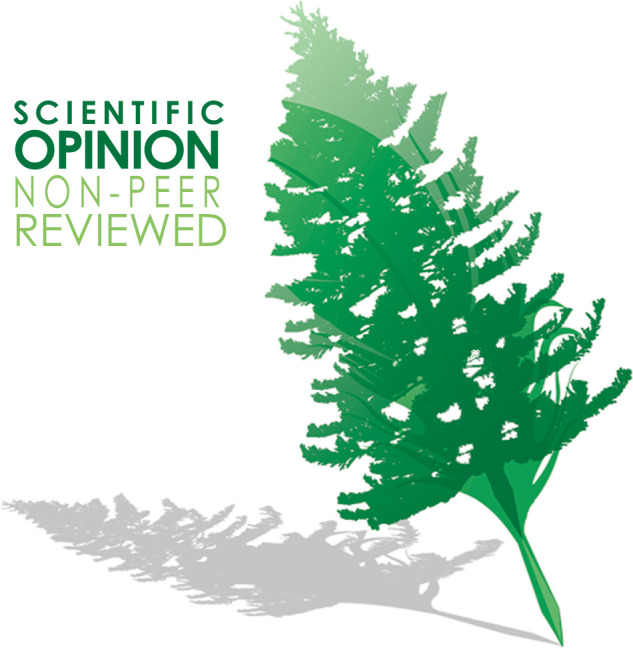

Over the 60 years of *Environmental Science & Technology*, the chemical landscape has expanded at an unprecedented pace. Growing from a few thousand in the 1960s (Figure 1a), the documented chemical universe now encompasses 200 million substances, with >20 million new chemicals entering the CAS Registry every year.^1^ Many chemicals undergo extensive transformation within biological systems and the environment. The resulting transformation products (TPs), many of which remain unknown and may be persistent, mobile, and/or toxic, frequently outnumber their parent compounds. Thus, the potentially relevant chemical space expands far beyond the substances produced, documented, and monitored. This presents a fundamental challenge when designing intervention measures: not only detecting chemicals in complex environmental and biological matrices but also understanding mixtures, transformations, and cumulative effects while considering shifting time lines and exposures (Figure 1a).

**Figure 1:**
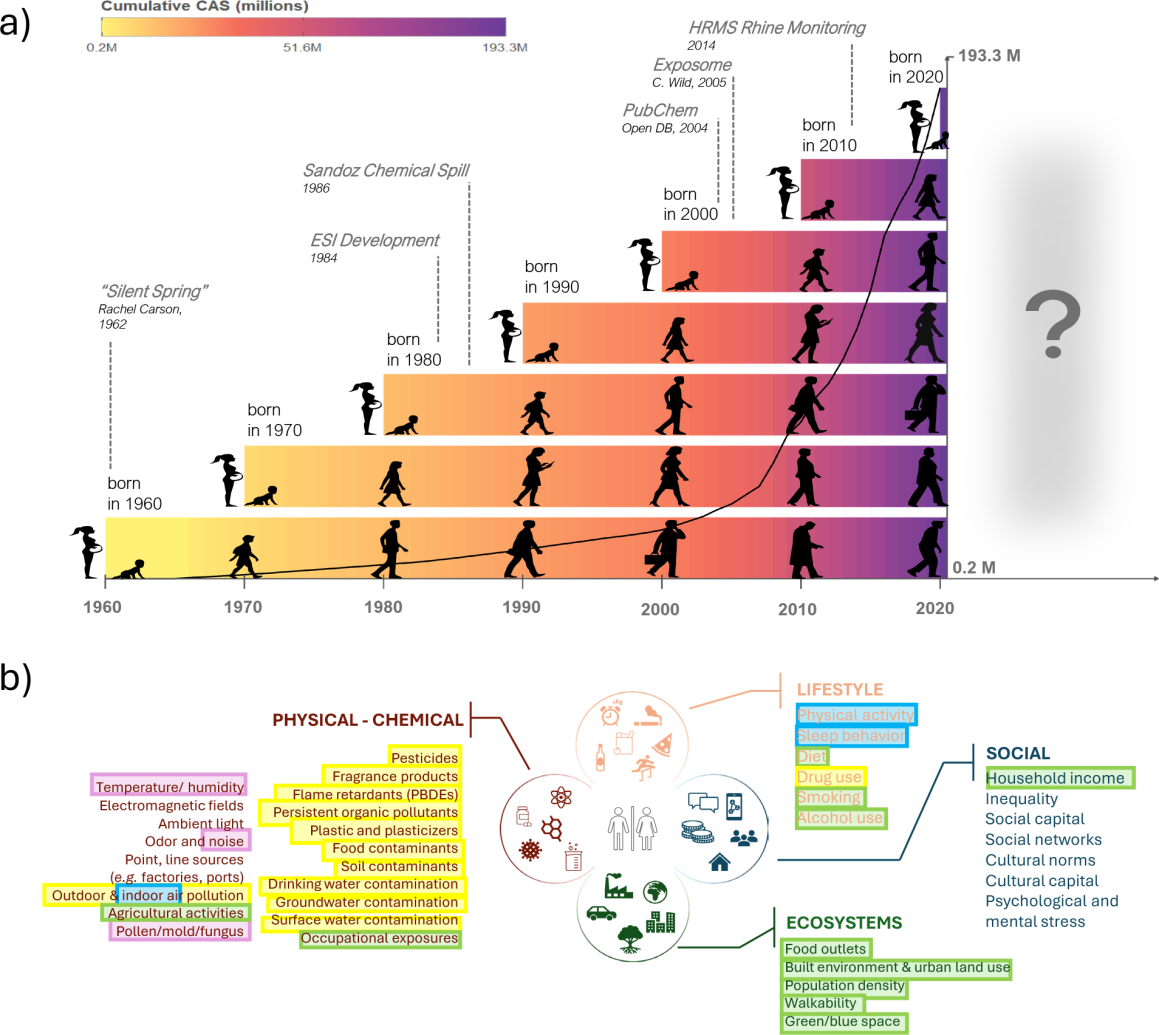
(a) Cumulative count of compounds in the CAS Registry between 1965 and 2021 (stripe shading and black line), with life trajectories starting each decade superimposed over the shaded stripes. Original data from ref 1. Major events discussed in this Viewpoint are indicated in the time line. (b) Exposome, modified from ref 2, with markup to indicate environmental factors that are now in reach for large scale exposomics efforts: yellow for factors that can be measured with high-resolution mass spectrometry (HRMS), blue for factors that can be measured with wearables, pink for factors that can be measured with other (non-HRMS) techniques, and green for factors derived from centralized environmental monitoring systems, geospatial modeling, or administrative data sources.

This is where open data and knowledge exchange are crucial. This Viewpoint takes a step back to see what is already possible and what is within reach and calls for the community to embrace open, collaborative, discovery-based exposomics research to help transform environmental analysis and exposomics into a new era in the coming decades.

Environmental science has evolved considerably in 60 years. *Silent Spring* (Figure 1a) raised global awareness of the environmental harm caused by indiscriminate chemical use. The 1986 Sandoz chemical spill stimulated daily Rhine monitoring (commencing in 1994–1995, upgraded to high-resolution mass spectrometry (HRMS) in 2014) to improve spill detection. This was facilitated by key advancements in mass spectrometry around the same time, including electrospray ionization (ESI) in 1984 (Figure 1a), which enabled broader detection of chemical space. However, another problem remained. Many unknown compounds were waiting to be discovered, while much chemical information was “closed”. A major breakthrough was the 2004 launch of PubChem, one of the first large scale, openly accessible chemical databases, enabling wider chemical data sharing and the development of new identification methods. Chris Wild introduced the “exposome” to complement the genome in 2005, enhancing efforts to understand environmental causes of diseases. This coincided with the commercial release of the Orbitrap, a key HRMS development. Together, these milestones marked the beginning of a new era, shifting environmental science toward an open, collaborative discovery-driven HRMS-based framework.

The reality is that discovery-driven exposomics is far beyond one research group and requires a concerted community effort to join forces and reveal the missing environmental components responsible for adverse effects. Reviewing the aspects of the exposome outlined in 2020,^2^ many of these are now a reality within reach (Figure 1b). In practice, this is established not only through analytical advancements such as HRMS but also by the integration of open science tools into exposomics workflows. Annotation workflows now combine compound and spectral matching using several open libraries, such as PubChem, the CompTox Chemicals Dashboard (CCD), PubChemLite, the Human Metabolome Database, MassBank, MassBank of North America, the Global Natural Product Social molecular networking (GNPS), and many more. By reporting and registering new compounds, the open chemical space has expanded, with PubChem growing to 123 million compounds. This progress has led to increased data generation, challenging both scientists and computational methods to handle all of the information. This in turn spurred the creation of smaller collections such as the CDD and PubChemLite to help constrain the exposomics search space to more relevant subsets, along with the development of suspect screening approaches and corresponding resources such as the NORMAN Suspect List Exchange to interrogate the data in new, more efficient ways.

Since several HRMS vendors are still used across laboratories, open source HRMS workflows are essential, providing vendor agnostic approaches to interrogate data. Tools such as mzMine, MS-DIAL, patRoon, and MassCube, operated in open software environments like R and Python, facilitate data processing and compound annotation. Progress does not stop there. *In silico* compound annotation approaches generally reliant on database lookup such as MetFrag, CFM-ID, and SIRIUS are now supported by *de novo* approaches such as MSNovelist, while molecular networking and MS2Query (among others) help identify analogues. TPs can be predicted with approaches such as enviPath and BioTransformer and screened using patRoon. FAIR-TPs facilitates community contributions of TPs from the literature to provide further data to help improve predictions. Finally, clearly communicating identification confidence helps exchange information about tentatively identified compounds,^3^ facilitating global comparison and cooperation.

How can open source exposomics work in practice? Talavera et al.^4^ applied a fully open science cheminformatics workflow to investigate environmental drivers of Parkinson’s disease penetrance in household dust samples. By integrating LC-HRMS instrumentation with open source computational tools and databases, and through collaboration between clinicians, exposomics, and metagenomics experts, statistically significant associations were identified between replacement and tire wear chemicals on disease penetrance, associations that are now being validated further. This highlights how collaborative open science efforts facilitate an improved understanding of complex biological systems.

While collaboration and sharing of resources are critical, another key to ushering in the next era of discovery-based exposomics research will be the transition into standardized, high-throughput, cost-effective settings. Increasing throughput and reducing cost will transition exposomics discovery into routine applications, similar to the standardization of untargeted metabolomics through specialized kits. A data-driven selection of exposome chemicals for high-throughput kit formulation is being prepared for release soon. Capturing the entirety of chemical space in an “all for one” exposomics method remains unrealistic. Instead, progress will depend on standardization of certain subsets of chemical space, embracing its complexity by breaking it up into reproducibly measurable pieces. This shift is essential for achieving the systemic characterization of environmental exposures and addressing the missing environmental component in disease, which represents not only a scientific gap but also a barrier to public health progress. Unlike genetics, environmental factors are often modifiable, offering substantial opportunities for meaningful public health intervention.^5^

The potential benefits of identifying actionable environmental drivers are enormous. The tools are largely in place. What remains is the collective will to integrate them. It is time to embrace open, collaborative, discovery-driven exposomics and allow the data to reveal where health and environmental intervention can achieve the greatest impact.
